# Innovative Approaches to Emergency Medical Services Fellowship Challenges

**DOI:** 10.5811/westjem.2019.10.43830

**Published:** 2020-02-21

**Authors:** Benjamin W. Weston, Joshua Gaither, Kevin Schulz, Saranya Srinivasan, Jennifer J. Smith, M. Riccardo Colella

**Affiliations:** * Medical College of Wisconsin, Department of Emergency Medicine, Milwaukee, Wisconsin; † University of Arizona, College of Medicine, Department of Emergency Medicine, Tucson, Arizona; ‡ Arizona Emergency Medicine Research Center, Tucson, Arizona; § Houston Fire Department, Houston, Texas; ¶ McGovern Medical School at the University of Texas Health Science Center at Houston (UTHealth), Department of Emergency Medicine, Houston, Texas; || Baylor College of Medicine, Texas Children’s Hospital and Department of Pediatrics, Section of Emergency Medicine, Houston, Texas

## Abstract

**Introduction:**

Since the development of an Accreditation Council of Graduate Medical Education (ACGME)-accredited emergency medical services (EMS) fellowship, there has been little published literature on effective methods of content delivery or training modalities. Here we explore a variety of innovative approaches to the development and revision of the EMS fellowship curriculum.

**Methods:**

Three academic, university-based ACGME-accredited EMS fellowship programs each implemented an innovative change to their existing training curricula. These changes included the following: a novel didactic curriculum delivery modality and evaluation; implementation of a distance education program to improve EMS fellows’ rural EMS experiences; and modification of an existing EMS fellowship curriculum to train a non-emergency medicine physician.

**Results:**

Changes made to each of the above EMS fellowship programs addressed unique challenges, demonstrating areas of success and promise for more generalized implementation of these curricula. Obstacles remain in tailoring the described curricula to the needs of each unique institution and system.

**Conclusion:**

Three separate curricula and program changes were implemented to overcome specific challenges and achieve educational goals. It is our hope that our shared experiences will enable others in addressing common barriers to teaching the EMS fellowship core content and share similar innovative approaches to educational challenges.

## INTRODUCTION

In 2012, the Accreditation Council for Graduate Medical Education (ACGME) approved an accredited fellowship in the area of emergency medical services (EMS).^1^ Along with this accreditation, curricular core content and competencies were identified to guide the education and training of EMS physicians.^2^ While prototype fellowship and residency EMS curricula have been previously outlined prior to EMS ACGME accreditation, there is little published material to guide effective content delivery or innovation in training modalities and to evaluate whether these changes and methodologies have been effective.^3^–^5^ Available guidance has emphasized the importance of creating a formalized curriculum that reflects core content in a diversity of educational formats.^6^

In creating a delivery model for the EMS fellowship curriculum, different institutions have taken modified approaches to best suit their individualized needs given the resources at hand. The EMS fellowship curriculum requires an average of three hours per week of planned didactic experiences, totaling over 150 hours of time per year.^1^ These didactics, often presented to a single fellow, can easily become dry and monotonous. For the first intervention, we discuss the incorporation of a variety of lecture, discussion, and training formats in the development of an interesting and dynamic didactic portion of the curriculum.

Additionally, ACGME-accredited EMS fellowships are mandated to provide fellows with an experience in rural EMS.^1^ Numerous demands on a fellow’s time coupled with low call volume in rural settings have limited the rural EMS experience. For the second intervention, we discuss distance-based efforts to improve rural EMS education for EMS fellows. Finally, although physicians of any specialty may pursue an EMS fellowship, most curricula assume fellows will have an emergency medicine (EM) background, which may leave gaps in clinical training and challenges in maintaining board certification for non-EM trained fellows. For the third intervention, we discuss the adaptation of a standard EMS fellowship curriculum to accommodate non-traditional specialties.

## METHODS

Outlined below are the methods undertaken for each of the three discussed interventions.

### Novel Didactic Curriculum

We conducted a before-and-after retrospective review, approved by our institutional review board, comparing grand rounds evaluation data from before and after the implementation of a novel EMS fellowship curriculum. At this institution, grand round presentations for the EMS fellowship are typically provided for three hours on Thursday afternoons. Following each presentation, each attendee completes an evaluation form scoring the presentation on effectiveness and value, context (applicability to EMS practice and boards), content (instructor expertise on information delivered), and tension (active learner engagement and level of instructor expertise). These are each evaluated on a scale of 1–9 with 9 being the highest score achievable. Overall score is the average of each of these categories. In the 2014–2015 academic year, a conventional, lecture-based curriculum was in place based on topics drawn from both lecturer experience and expertise as well as from the core content required for EMS fellowship.

During the 2015–2016 academic year, instead of traditional didactic sessions delivered by an EMS core faculty using PowerPoint slides, we developed a novel approach to grand rounds by implementing thematically-focused weeks consisting of a combination of experiential-focused lectures (such as an individual lecturer’s experience managing a mass gathering event); system-specific topics (such as continuing education processes in our regional EMS system); chapter-focused discussions; case discussions; journal clubs; and special events. Special events included hands-on training modules, procedural skill practice, and interactive-lecture formats with medical students and residents.

A variety of instructors, ranging from core faculty and content experts to local providers and EMS fellows, were incorporated. Examples of the modified novel didactic curriculum are provided in Table 1. Data from attendees who completed presentation evaluations for EMS grand rounds presentations from the 2014–2015 “conventional” curriculum and the novel 2015–2016 curriculum. Linear mixed models accounting for random lecturer effect were used for post- vs pre-intervention comparisons.

### Distance-Based Tool for Rural Engagement

In 2016, while expanding the EMS fellowship complement from one to two fellows per year a distance-learning platform was implemented allowing EMS fellows to participate in the fellowship didactic curriculum when off-site, at a rural EMS location (Table 2). This project addressed two problems: 1) the need to provide more time for fellows at rural EMS locations; and 2) the need to prevent EMS clinical (field time) overlap between fellows. By implementing this distance education program, on didactic days the EMS fellows could both participate in didactic experiences while operating clinically in different geographic regions (one physically located at their rural EMS clinical site while the other was at their primary urban site). After completing didactic requirements, the rural fellow was able to spend the remainder of the day interacting on-site with the rural crews.

To implement this project, existing equipment and programs were used including the Panopto video platform (Panopto, Seattle, WA, Version 5.4.0), a web-based system supporting a live webcast including lecture slides with audio and video transmission to the distance site. The live webcast was supplemented by real-time discussion along with question-and-answer sessions between the broadcasting location and the rural site using a social media platform Convo (Convo.com, Los Altos, CA). Both systems were available free of charge from the hosting institution and could be run from a computer or a mobile device. To improve audio quality a microphone was purchased at a cost of less than $150.

### Approach to a Non-Emergency Medicine-Trained Fellow

A similar approach was used in development of the curriculum for two non- EM trained fellows: one from a pediatric background and one from an anesthesiology background. The curriculum included identifying gaps in the non-traditional fellows’ knowledge, addressing those gaps using customized supplemental experiences in the field or in the emergency department (ED) setting as appropriate, providing personalized oversight and support from fellowship faculty and supervising medical directors, and developing a plan to address the fellows’ maintenance of their primary board certification (Table 3).

Once gaps in knowledge and skills were identified prior to the start of the academic year, novel processes and experiences were developed to address these gaps. To address one fellow’s concern about inexperience in management of critically ill adults, an “Adult EM Boot Camp” was developed including target, high-volume ED shift exposure while paired with EMS educational faculty, specific training on specialized areas such as electrocardiogram interpretation and cardiovascular care, and discussions on management of critical and non-critical patients. As additional measures to enhance clinical education, the fellowship didactic curriculum was front-loaded with topics identified as knowledge gaps, fellows attended the affiliated EM residency’s didactic conference when appropriate, and fellowship faculty used cadaver labs and simulation to address gaps in knowledge and experience in the non-traditional EMS fellows. The fellows were also given assignments to complete within the scope of their medical direction responsibilities outside of their core specialty, such as the pediatric-trained fellow focusing on adult-oriented projects.

When operating in the field setting, the non-traditional fellows were provided modified oversight and support by the EMS faculty and supervising medical directors. During field operations with faculty, educational conversations and didactic sessions were geared toward knowledge gaps while emergency calls were preferentially selected as those likely to fill a gap in knowledge or experience for the fellow. Additionally, tapes of direct medical oversight interactions were reviewed with the fellows, especially for patients outside of the fellows’ previous training.

Finally, accommodations were made to allow for the maintenance of primary board certification for non-EM fellows. Intradepartmental agreements were made to allow for clinical work in their area of primary board specialization, while maintaining duty-hour and fellowship requirements.

## RESULTS

With the introduction of the novel didactic curriculum, a total of 537 evaluations were completed and evaluated for 115 distinct lectures between the before-and-after periods. The before (conventional) period consisted of 210 completed evaluations for 54 distinct lectures and the after (novel) period consisted of 327 completed evaluations for 61 distinct lectures. Significant improvements in the after group as compared to the before group were noted in the categories of effectiveness and value, content, tension, and overall score (Table 4). No significant difference was noted in the category of context.

Using the distance-based rural curriculum, 48 lecture sessions were delivered over the course of the 2016–2017 academic year. On three occasions (6.25% of sessions) technology issues prevented successful delivery of the didactic curriculum. Didactic material was successfully presented from both the primary EMS fellowship site and the distance or rural EMS site. Fellow time at the rural EMS site doubled from five hours per day to 10 hours per day (Table 5). In addition, the fellow was able to be present for crew change, doubling the number of EMS providers he or she had contacted with for the day. Additionally, prior to this intervention EMS provider continuing education (CE) was widely available but required off-shift participation and was limited to one on-site lecture per year. After this intervention, rural EMS providers had access to an expert physician and more than 60 hours of annual EMS-provider CE available on site with optional on-shift participation.

In the case of the pediatric EM/EMS fellow, not only did the fellow express confidence in performance of the skills and tasks required of an EMS fellowship graduate and EMS physician, but the fellow subsequently passed her American Board of Emergency Medicine (ABEM) EMS subspecialty board exam on her first attempt. The anesthesia/EMS fellow continues his fellowship at the time of this writing and has successfully transitioned to taking independent calls with faculty oversight in specific cases and feels confident in performing his fellowship duties after the modified fellowship orientation. He ranked among the top scores on the in-service EMS board exam and sat for the EMS subspecialty boards in 2019, the results of which are expected to further validate the process.

## DISCUSSION

As a relatively new ABEM board subspecialty, EMS fellowships continue to develop and identify best practices and strategies to overcome common training program barriers. Working within EMS systems and with individual educational institutions may present both opportunities and challenges to fellowship programs. Given the large variability of EMS system structures, practices, and resources across the country, individual fellowships must be able to build on system strengths and develop innovative solutions for system challenges. We presented three such approaches to innovate within the structure of the EMS fellowship to maximize learning for fellows. We believe that these approaches have applicability to many different fellowship programs across the country.

While literature on best practices and innovative approaches to the EMS fellowship is sparse, fellowships from other specialties may serve as guides to how to overcome the didactic, distance, and knowledge gap challenges faced by our three described programs. From a general curriculum design perspective, the radiology fellowship at Emory University School of Medicine has described its efforts to develop a multifaceted didactic curriculum that involves a variety of educational formats to engage learners beyond the traditional lecture.^7^ In addressing distance-education based challenges, several surgery fellowships have shared programs designed to enhance rural and international experiences while maintaining strong core content.^8^,^9^ Likewise, a distance-based educational program has been developed into its own fellowship for general practice doctors in India to allow for a supportive and engaging learning environment during the early years of practice.^10^ Faced with gaps in clinical knowledge among residency graduates, a hematology oncology fellowship developed interactive, cadaveric, and simulation-based workshops to prepare trainees for the fellowship experience.^11^

As these and other innovations are implemented and evaluated within any fellowship, it is important to maintain an overarching goal of pursuing best practices. First, specific to the EMS fellowship, one must optimize the fellow’s experience and education by ensuring a variety of experiences and opportunities across the spectrum of prehospital care. Second, one must improve the fellow’s clinical exposure in both controlled and uncontrolled settings, using experiences both on scene and during transport, in addition to experiences in the ED as appropriate. Lastly, the fellows’ procedural skill competency and teaching skills must be improved through both hands-on experiences and by instructing other learners such as paramedics. Achieving these best practices in fellow education can be at times challenging given resource limitations and the various clinical, financial and political implications of fellow participation in each unique EMS system.

Several limitations were present with each of the discussed interventions. While we did note improved ratings for the novel didactics presented in the first intervention, the limited number of one to two fellows per year made it difficult to evaluate actual learner outcomes and the extrapolation of results to educational importance or performance outcomes proves challenging. With the distance-based rural curriculum, technical challenges occurred that resulted in at times difficult communication. Additionally, the increase in the EMS fellow compliant from one to two fellows may have confounded the improved relationship with the rural EMS agency due to increased physician exposure. In the training of the non-EM trained fellow, quantitative results were difficult to attain given the limited number of fellows. In all of the interventions, the creation and implementation of modified curricula may be time and resource intensive for some fellowship programs.

In this report, we highlight three novel approaches to modify EMS fellowship curricula to overcome barriers while maintaining educational goals and providing optimal fellowship experiences. While each addresses a specific area of the fellowship–creating an engaging and diversified didactic curriculum; developing a distance-based tool for rural EMS education; and modifying a curriculum to train a non-EM trained fellow–we believe that these modifications are widely applicable to other fellowship programs facing similar issues. The interventions put in place were not unique to our institutions, but rather were common to most academic EDs and could therefore be implemented at other programs facing similar challenges. While we recognize that each fellowship program faces a unique set of challenges and resources may vary potentially limiting the broad applicability of our approaches, we hope that our experiences can inform other fellowships.

## CONCLUSION

We have presented interventions in which three separate EMS fellowship programs across the United States developed different, successful models to overcome specific challenges and achieve educational goals. We believe that these issues are generalizable and potentially faced by other EMS fellowship programs and may aid in overcoming similar challenges. We hope that others will share similar experiences, thereby encouraging the development of best practices for educational curriculum and innovative approaches to EMS fellowships.

## Figures and Tables

**Table 1:** Novel didactic curriculum examples.

Format	Examples
Experiential focused	Scene Safety and Size Up Just Culture Model in EMS Delivering an Effective Presentation
System specific	Education in Milwaukee County EMS Wisconsin Disaster Preparedness History of Milwaukee County EMS
Chapter focused	Interfacility Transportation Ambulance Safety Medical Management of Mass Gatherings
Case discussions	Public Relations Case Review UW Madison Football Crush Complications in Air Transport
Journal clubs	Point of Care Ultrasound in EMS SALT Triage Treating Confined Space Injuries
Special events	Emergency Vehicle Operations Course Physician Base Training Trauma Stabilizing Procedure Practice

*EMS*, emergency medical services.

**Table 2:** Key Elements of Distance-Based Tool for Rural Engagement.

Key elements
Located at rural site On-scene rural emergency medical services (EMS) care Web-based live lecture engagement Enhanced rural EMS provider continuing education Social media-based question and answer sessions

* Linear mixed models accounting for random lecturer effect were used for post versus pre intervention comparisons.

**Table 3:** Key elements of training the non-emergency medicine (EM) fellow.

Key elements
Identify gaps in knowledge and procedure skills On shift training with EM faculty Front load didactic curriculum with knowledge gap topics Utilize existing grand rounds topics as applicable Implement cadaver and simulation experiences Choose field responses and online medical control reviews strategically to address gaps Accommodate time for maintenance of primary board skills

**Table 4:** Fellow schedule at rural emergency medical services location before and after distance education intervention.

Category	Total (n=537)	Before (n=210)	After (n=327)	P Value*
Effectiveness and value	6.7 (1.4)	6.4 (1.3)	6.9 (1.5)	<0.001
Context	6.9 (1.4)	6.9 (1.2)	6.9 (1.5)	0.508
Content	6.9 (1.4)	6.6 (1.3)	7.1 (1.4)	<0.001
Tension	6.8 (1.2)	6.6 (1.2)	6.9 (1.2)	0.001
Overall	6.8 (1.2)	6.6 (1.1)	7.0 (1.2)	<0.001

**Table 5:** Key elements of training the non-EM fellow.

Key elements
Identify gaps in knowledge and procedure skills On shift training with EM faculty Front load didactic curriculum with knowledge gap topics Utilize existing grand rounds topics as applicable Implement cadaver and simulation experiences Choose field responses and online medical control reviews strategically to address gaps Accommodate time for maintenance of primary board skills
